# Tracers in Gastric Cancer Surgery

**DOI:** 10.3390/cancers14235735

**Published:** 2022-11-22

**Authors:** Zhiyan Li, Xianghui Li, Xudong Zhu, Shichao Ai, Wenxian Guan, Song Liu

**Affiliations:** Department of Gastrointestinal Surgery, Nanjing Drum Tower Hospital, The Affiliated Hospital of Nanjing University Medical School, Nanjing 210008, China

**Keywords:** gastric cancer, gastrectomy, tracer, blue dye, indocyanine green, carbon particles, radioactive tracer

## Abstract

**Simple Summary:**

Gastric cancer is a major health risk, and surgery is the primary curative option. However, the appropriate extent of lymph node dissection and appropriate surgical margins are two major issues that need to be addressed. Clinical tracers are an excellent solution. Therefore, we compiled a list of common clinical tracers for gastric cancer surgery to help surgeons better select and use them.

**Abstract:**

The treatment of gastric cancer mainly depends on radical gastrectomy. Determination of appropriate surgical margins and adequate lymph node (LN) resection are two major surgical steps that directly correlate with prognosis in gastric cancer. Due to the expanding use of minimally invasive procedures, it is no longer possible to locate tumors and LNs through touch. As an alternative, tracers have begun to enter the field due to their capacities for intraoperative visualization. Herein, we summarize the application of contemporary tracers in gastric cancer surgery, including isosulfan blue, methylene blue, patent blue, indocyanine green, carbon particles, and radioactive tracers. Their mechanisms, administration methods, detection efficiency, and challenges, as well as perspectives on them, are also outlined.

## 1. Introduction

With an estimated annual incidence of more than 100 million cases, gastric cancer (GC) is the fifth most frequent cause of cancer globally [1]. It is the fourth leading cause of cancer deaths for both sexes, accounting for 7.7% of all malignancy fatalities [1].

Nowadays, radical surgery is still the most effective treatment for GC. The most critical issues in radical GC surgery are thorough lymph node dissection and the determination of surgical margins [2]. Suitable acquisition of LNs is significantly connected to pathological staging and prognosis [3,4]. However, for most patients with early GC without LN metastases, biopsy of sentinel lymph nodes (SLNs) for the detection of metastases to determine extended or limited resection appears to be the most appropriate option [5]. Additionally, despite the widespread application of laparoscopy, it is difficult to distinguish the tumor site and identify resection lines due to the inability to palpate through the stomach wall [6].

Tracers are useful for addressing the aforementioned issues during gastrectomies and lymphadenectomies. The application of blue dye, indocyanine green (ICG), carbon particles, and radioactive tracers in GC surgery has been explored. Through specific deposition in LNs or staining of tissue surrounding the tumor, the surgical target can be displayed more clearly. For more precise detection, dual tracers are implemented in surgery, in which the simultaneous application of blue dyes and radioactive tracers when harvesting SLNs is most frequently used [7].

We aimed to include tracers that are being employed in clinical GC surgery. Their mechanisms of action, application methods, and clinical effects are summarized. Future perspectives are also offered in this review.

## 2. The Current Status of GC Surgery

GC is a carcinoma with a high propensity for LN metastasis. A total of 87.5% of patients with local recurrence after radical resection show manifestations as LN metastases [8]. Traditionally, extended LN resection (D2) has been considered a radical method for LN dissection in advanced gastric carcinoma. The number of detected LNs is closely related to pathologic staging and prognosis [4]. The eight edition of the classifications of the International Union against Cancer/American Joint Committee on Cancer recommended the dissection of more than 16 LNs for the exact TNM staging of GC, while Japanese Regulations on the Management of Gastric Cancer recommend that at least 15 LNs should be dissected [9,10]. According to the latest version of the German S3-Leitlinien, the number of LNs required for detection in a D2 lymphonodectomy is up to 25 [11]. However, for patients with early-stage GC without LN metastases, especially patients suffering from cT1N0 GCs smaller than 5 cm in size, D2 lymphadenectomy is redundant and can potentially adversely affect the prognosis [12,13,14]. The concept of SLN biopsy has been put forward, recommending the biopsy of the first LN that receives lymphatic flow and in principle bears the greatest transfer risk to predict whether other LNs have metastasized and determine the follow-up operation plan [15]. This approach reduces patient injury and aids recovery. After further development, the notion of the sentinel lymphatic basin, where detected SLNs and the majority of metastatic LNs are located, was developed to overcome the complicated and multidirectional lymphatic drainage system and the high incidence of skip metastases in gastric cancer [16,17,18]. Tracers implemented intraoperatively can effectively assist in LN detection as needed, whether to discover as many LNs of patients with advanced GC as possible or accurately identify SLNs of patients in the early stage [19]. Additionally, in a gastrectomy, complete resection with an appropriate margin is necessary. Safe proximal resection margins can improve curability and reduce recurrence [20]. According to the Japanese GC surgical standards, the resection margin ought to be at least 2 and 3–5 cm for patients suffering from early GC and advanced GC, respectively [10]. Due to the difficulty of touching the stomach wall, it is arduous to diagnose tumor location and define the resection lines during laparoscopic surgery, especially when a tumor is located in the upper third of the stomach [6,21].

Currently, several methods are employed for tumor margin recognition, such as metal clips and intraoperative endoscopy [22,23]. The time-consuming nature of the procedures, possible complications, and the need for additional personnel and equipment are bottlenecks for these methods. In this context, tracers have gained attention for their simplicity and low cost [6]. In the stomach, there is a complicated and multidirectional lymphatic system. The lymph from the stomach wall drains into lymphatic vessels arising in the mucosa, which subsequently drain into the peri-gastric lymphatic system spread along the main arteries of the stomach [24]. For non-selective tracers, proper particle size is the basis of tracing. Since the pore width between the capillary lymphatic cells is about 100–500 nm, which is much larger than that of the capillary endothelium, a tracer with suitable particle size injected around the tumor can selectively enter capillary lymphatic vessels and perform the role of lymphatic tracing [25]. Moreover, annexin A 1, sialyl-related antigen, and E-cadherin have been reported to be overexpressed in metastatic LNs [26]. Regrettably, no clinical tracer has yet exploited these molecules as targets to increase the accuracy of tracer targeting. Currently, blue dyes, ICG, carbon particles, and radioactive tracers are increasingly employed in GC surgery.

## 3. Tracers in GC Surgery

### 3.1. Blue Dyes

**Figure 1:**
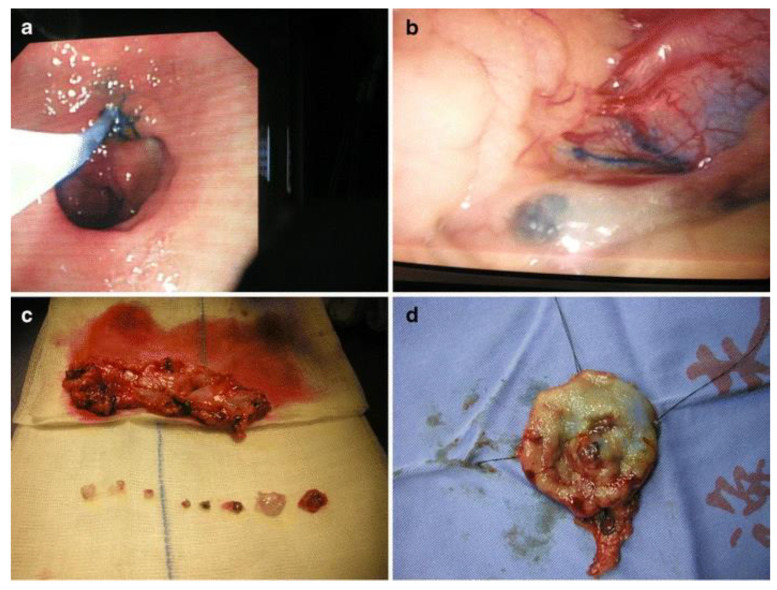
Procedure for laparoscopic local resection with sentinel node biopsy. (**a**) Isosulfan blue was injected into the peritumoral submucosal layer after the lesion was localized through an intraoperative endoscopy. (**b**) Within 5 min of dye injection, lymphatic flow from the lesion was visible. (**c**) Sentinel nodes were isolated, while perigastric tissue was extracted. (**d**) Local resection lines were marked at the four edges of the resection margin. Reproduced with permission [33]. Copyright 2008, Springer Nature.

Blue dyes, including isosulfan blue, patent blue, and methylene blue, are extensively applied in SLN biopsies. They are popular due to their inexpensiveness, wide availability, and tractability [27]. They possess the capacity for lymphatic targeting owing to the binding of their sulfonic acid group to serum proteins [28,29]. This characteristic ensures that they have a suitable particle size to pass through lymphatic vessels while trapped in LNs [30]. However, methylene blue, as a substitute for these dyes, does not bind to plasma proteins because of the lack of sulfonic acid groups [31]. Its LN specificity has therefore been revealed to be inferior [28]. Through visual inspection without any special equipment, the tumor borders and the flow of lymphatic vessels deriving from the GC site can be monitored intraoperatively after peritumoral injections (Figure 1). LNs are thus visualized, which can be beneficial to SLN harvesting or extensive LN dissection, as needed. Appropriate waiting time is required between injection and detection, as SLN staining takes approximately 5 to 10 min, and second-echelon LNs will be stained as time progresses [32]. These dyes still have several drawbacks, such as difficulty in quantification and potential allergic reactions [27]. The rapid transit of dyes through lymphatic vessels after injection, as well as the interference of thick adipose tissue, can possibly allow SLNs to be omitted or multiplied [5].

#### 3.1.1. Isosulfan Blue

As a triphenylmethane-based rosaniline dye, isosulfan blue was the first dye approved by the Food and Drug Administration (FDA) for lymphangiography due to its superior properties and low number of adverse reactions [34]. Endoscopic submucosal injection or direct serosa injection after laparotomy are the major methods for its clinical application [35]. One milliliter of one-percent isosulfan blue is implemented as a mainstream dose for imaging, which is usually injected at four spots around the tumor submucosally [36,37]. Different injection sites are accompanied by distinct imaging times. If the injection is subserosal, 5 min ought to be enough for it to flow into the SLNs, while the waiting time varies from 5 min to 15 min if a submucosal injection is performed [35,36,37,38,39]. The detection rate for isosulfan blue is as high as 90% or even 100%, with the average SLN number around three and the average number of dissected LNs being over 25 [37,38,40]. Possessing a positive predictive value of over 72%, a negative predictive value of around 87%, and a sensitivity of approximately 97%, isosulfan blue exhibits great potential as a tracer [36,38,41]. Nevertheless, the false-negative rate remains high, most likely as a consequence of the more convoluted lymph drainage or the disconnection of lymphatic vessels resulting from tumors larger than the T2 stage [41]. The indications of isosulfan blue need to be further explored.

#### 3.1.2. Patent Blue

Patent blue possesses the capacity to label tumors in situ and aid in the identification of LNs. The commonly utilized administration is an injection of 0.2 mL of 2% patent blue solution into four quadrants surrounding the tumor through an intraoperative gastroendoscopy [42,43,44]. Higher doses are required in subserosal or intramuscular approaches [45,46]. As reported, the dye is visible on the stomach serosa surface immediately after submucosal injection [16]. It takes 1 to 16 min to observe SLNs after subserous injection [47]. Stained LNs are still plainly visible until two hours later [48]. The detection rate is between 76.3% and 97.4% [45,47]. The particularly low detection rate in the study conducted by Rabin et al. [45] was probably due to the inclusion of too many patients with T3 tumors. The dosing method may have been another influencing factor. Although a small sample study illustrated that there existed no significant difference between submucosal and subserosal injections [47], studies adopting subserosal injections yielded worse data [46]. Along with LN detection, patent blue has been applied to ensure proximal surgical margins. With the guidance of clips placed using preoperative gastroendoscopy, patent blue dissolved in sodium hyaluronate has been injected submucosally during surgery, which guaranteed negative proximal resection, although some margins were less than the estimated length [21].

#### 3.1.3. Methylene Blue

An alternative to isosulfan blue, methylene blue is more readily available [49]. The methods and the doses of injections vary across studies. Intraoperative injection of diluted methylene blue solution into the submucosa, subserosa, or left and right gastric arteries has been attempted [50,51,52]. The submucosal injected methylene blue reached SLNs within 5 to 10 min [52]. SLN detection rates were high for each mode of administration, with a minimum of 87% [50]. However, the harvesting effect was relatively low in malignancies diagnosed as pT3 or pT4, partially due to the disordered lymphatic drainage [50,51]. It has been reported that the average number of LNs detected with the assistance of methylene blue was 36 [50]. It was especially effective for the identification of small LNs smaller than 6 mm in diameter [50]. Furthermore, methylene blue has been applied in picking out LNs after radical lymphadenectomy for more accurate N staging [53].

### 3.2. ICG

**Figure 2:**
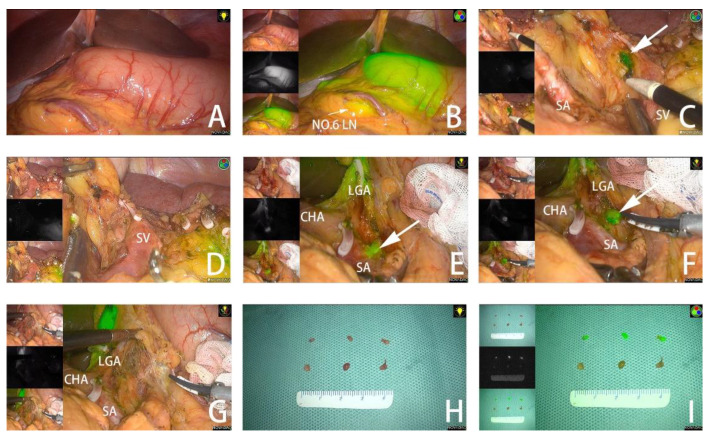
(**A**,**B**) Tumor observed under white light in fluorescent mode. (**C**) Fluorescent no. 10 LNs detected in fluorescent mode. (**D**) No remnant no. 10 LNs are found after dissection. (**E**) Fluorescent no. 11P LNs adjacent to splenic vessels. (**F**) No. 11P LNs separated from blood vessels. (**G**) No remnant No. 11P LNs are found after dissection. (**H**) LNs dissected from the specimen under white light. (**I**) LNs dissected from the specimen in fluorescent mode. The arrow points to the fluorescent LN. SA, spleen artery; SV, spleen vein; CHA, common hepatic artery; LGA, left gastric artery. Reproduced with permission [63]. Copyright 2022, Frontiers Media.

Analogous to blue dyes, ICG tends to attach to plasma proteins or bilirubin and is instantly absorbed by lymphatic vessels [32,54]. In addition to visual inspection, ICG has the capacity to facilitate infrared and fluorescence observation, making it a novel favorite among tracers. ICG, as a fluorescent dye with an excitation wavelength of around 800 nm, can shift its maximum absorption wavelength to 805 nm if combined with plasma proteins [55]. When exposed to light approaching the maximum absorption wavelength, the ICG-injected region absorbs the light and darkens, while the background portions reflect light and become brighter [17]. Tissues stained by ICG, even with a depth up to 5 mm, can hence be observed under infrared light [56]. To improve the visibility of the dye within human tissue, near-infrared fluorescent imaging (NIFI), infrared ray electronic endoscopy (IREE), and infrared ray laparoscopic system (IRLS) devices have been developed, which are compatible with minimally invasive surgery [57,58,59]. Furthermore, ICG exhibits a maximum fluorescence wavelength of 830 nm after photo-stimulation. Novel fluorescence imaging (FI) systems, including the Hyper Eye Medical System, D-light P System, and PINPOINT Endoscopic FI System, have been exploited to receive light around the wavelength of maximum fluorescence under conventional white light [13,17]. The observation results for intra- and post-operative fluorescence are illustrated in Figure 2. There is consensus that the observation efficiency of ICG using infrared light is much higher than that of ordinary light [60]. IREE and IRLS appeared to be more sensitive than FI systems [61], and IREE increased the sensitivity and recognition rate compared to NIFI [62].

ICG is favored because of its hypoallergenic potential, deep detection depth, high sensitivity, and stable signal [27]. The dose and timing of administration have been the subject of several scientific investigations. In recent research, submucosal injections at concentrations of 0.625 or 1.25 mg/mL were the most popular [2,63,64,65]. According to subgroup analysis, surgeries utilizing ICG with a concentration of 0.5 or 0.05 mg/mL had a higher pooled sensitivity than the 5 mg/mL subgroup [61]. Furthermore, the dosage depended on the equipment due to their different sensitivity to light. With the upgrade from the da Vinci Si to the more advanced da Vinci Xi or the PINPOINT system, the concentration of ICG has been halved as well [66]. When it comes to ICG at concentrations of 0.625 or 1.25 mg/mL, submucosal administration in four quadrants around the tumor, with 0.5–0.6 mL in each quadrant, is the most common approach [2,55,63,64,67]. Dosing from the serosal surface [68,69], along the greater and lesser curvatures of the stomach [70], or submucosally with gastrotomy incision [71] have been exploited. Chen et al. [68] conducted research to investigate the difference between submucosal and subserosal administration. There was little difference except for the lower cost and higher satisfaction scores in the subserosal approach group since no additional endoscopy was required. A gap of at least 20 min between drug administration and lymphadenectomy appeared to be more effective than immediate imaging [61]. The longest reported waiting time was up to three days, without increasing the dosage [72,73]. In general, there is still no unified criterion for dosage, which needs to be further standardized.

With the assistance of ICG, the SLN detection rate was able to reach 100% in several studies [56,74,75]. Regardless of the accuracy, sensitivity, specificity, positive predictive value, and negative predictive value, most rates were reported to be greater than 90% [73,75,76]. The accuracy and specificity were determined by clinical neoplasm staging [72,77]. Tajima et al. [72] found that the mean number of detected SLNs after injecting 1–3 days before surgery averaged more than 9, which was distinguished from averages of roughly 3 to 5 reported in other studies [74,78,79,80]. The small hydrodynamic diameter of ICG was possibly responsible for this finding, which led to the migration of ICG to second-tier nodes [81]. A nanocolloid-adsorbed ICG has been developed to increase its hydrodynamic diameter [80]. It has been pointed out that the application of ICG during gastrectomy and lymphadenectomy increased the total number of detected LNs, shortened the operation time, reduced blood loss, and lowered noncompliance [4,64,65,67,70]. Even if produced scarring after endoscopic submucosal dissection, the use of ICG still yielded positive effects [66]. However, it remains controversial whether ICG can benefit LN dissection after neoadjuvant chemotherapy-induced local histological changes [3,69]. Since fluorescence can be quantitatively analyzed to reduce human subjective error, research has been carried out to determine appropriate fluorescence values for SLN detection [82]. As expected, the fluorescence intensity of ICG-positive LNs was stronger compared to ICG-negative ones [56]. The threshold for SLN detection ought to be 10% of maximum signal intensity [83]. Nevertheless, in a prospective study, the false-negative rate was up to 46% following the preliminary study and remained at 14% even after additional green-stained node sections were examined through paraffin sectioning [84]. Visual inspection of the LNs may have contributed to this result. Obesity may lead to failed LN identification and prolonged operation times [57,74,85]. It has also been found that the staining appeared to be less associated with metastasis [86].

Preoperative submucosal injection of ICG has been employed in resection margin determination. ICG imagining brought about shorter operation times, less operative hemorrhaging, reduced hospital stays, and fewer positive margins [2,87,88]. More interestingly, resin-conjugated fluorescent indocyanine green-equipped endoscopic marking clips have been developed to mark margins [89]. ICG has also been used in real-time intraoperative vascular monitoring [90,91].

### 3.3. Carbon Particles

**Figure 3:**
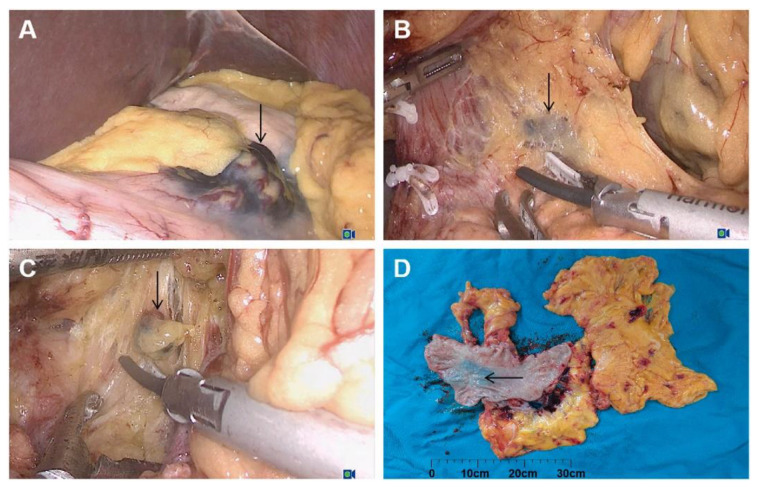
Carbon nanoparticle-labeling helped to find the lesion site (gastric angle) (**A**) and in draining lymph nodes (**B**,**C**) in laparoscopic radical gastrectomy. (**A**) Gastric angular lesions labeled with carbon nanoparticles; (**B**) black staining of lymph node no. 8a; (**C**) black staining of lymph node no. 11p; (**D**) gross specimen. Reproduced with permission [94]. Copyright 2021, Elsevier.

By blackening the serosa and staining LNs, carbon particles have been widely utilized for tumor localization and LN detection (Figure 3) [19,92]. The lymphatic targeting mechanism of carbon particles differs from the above-mentioned tracers. With an average diameter of 150 nm, carbon nanoparticle suspension injection (CNSI) tends to be administered through lymphatic capillaries rather than capillaries because the lymphatic capillary intercellular space is between 100 and 500 nm [25]. Furthermore, because of its diameter, CNSI engulfed by macrophages and aggregated in LNs migrates slowly, remaining in the SLNs for long enough without moving to higher-tier nodes [93].

Clinically implemented carbon particles have undergone a multi-stage transition from Indian inks and activated carbon to the latest CNSI. They are frequently utilized due to their ease of application, low cost, and safety [95]. Indian ink and activated carbon have been adopted in the clinical determination of surgical margins. It has been revealed that injecting activated carbon into the muscle layer or Indian ink into the submucosal layer before surgery ensured the identification of the location of the tumor on the serosal surface during surgery [92,96,97]. Injection times from 1 to 14 days before operations could be selected [92]. Although all margins were guaranteed to be pathologically negative, proximal resection margins were shorter than expected in 21% of patients, especially in patients with poorly differentiated or signet ring cell lesions [92]. Intraoperative endoscopy thus remains the most accurate approach to confirm resection margins, and defining tumor boundaries with carbon particles is still a long way off.

CNSI is the most suitable type of carbon particle for LN detection. Appropriate submucosal administration times are reported to range from 6 h to 2 days [93,94]. A waiting time of less than 6 h might lead to poor imaging effects, while injecting 1 to 2 days before surgery showed similar efficiencies [98]. For the injection method, CNSI (1 mL: 50 mg) is usually dosed at four points submucosally or at five points subserosally [94,99]. The no. 12b LN is another target spot for administration to trace para-aortic LNs [100]. It has been shown that CNSI-assisted lymphadenectomy possessed the advantages of shorter operation time, less bleeding, and more detected LNs [94,98]. In comparison to the groups adopting methylene blue or ICG, the number of LNs found using CNSI was considerably higher [100,101]. Additionally, CNSI has been found to be beneficial for identifying positive metastases, synchronous multiple GC, and micro-LNs [94,99,101]. In the study conducted by Yang et al. [25], the influence of neoadjuvant chemotherapy on the accuracy of N staging was investigated. Injecting CNSI before neoadjuvant chemotherapy favored the diffusion of CNSI in LNs, which could theoretically improve staining and help in tracing as many LNs as possible. To conclude, carbon particles are excellent clinical tracers with promising development prospects and have been extensively studied.

### 3.4. Radioactive Tracers

Many species of radioactive tracers have been employed in clinical LN tracing. Technetium-99m (99mTc) antimony sulfur colloid, 99mTc tin colloid, and 99mTc sulfur colloid are preferentially selected [102]. Applications of Indium-111 penetreotide and positron-emitting gallium-68-DOTA-peptides in surgeries of gastroenteropancreatic neuroendocrine tumors have also been reported [103]. Similar to carbon nanoparticles, radioactive tracers tend to migrate to the SLNs within 2 h and stay there for more than 20 h thanks to the phagocytosis of macrophages [104]. As a metastable isomer, 99mTc has a half-life of about 6 h and emits rays of 140 keV in LNs [32]. Signals are identified by gamma probes for LN detection. Due to the large particle size of the tin colloid, which allows a longer staying time inside SLNs to reduce the false-negative rate, it has been well-received [105]. However, the 99mTc sulfur colloid seems to be more effective than the 99mTc tin colloid [106]. The objective measurement of radioactivity, the ability to identify SLNs even in dense intraperitoneal adipose tissue, and the comparatively lengthy LN retention time are benefits of radioactive tracers [13]. Drawbacks, such as expensiveness, radioactivity, lack of visual examination, challenging lymphatic vessel and pool identification, and strong background scattering, interfere with their application [5,27].

The 99mTc radioisotope tracers were empirically injected submucosally into the surroundings of a lesion 1 day before surgery [107]. Injecting 2 mL of radioactive tracers into four quadrants is a common method [108,109,110]. There are also application examples of intraoperative injections [111,112]. Commonly, if the radioactivity level of an LN is ten times higher than the background, it is defined as a hot LN [110]. The detection rate, sensitivity, and specificity of LN tracing using radioactive substances alone have been reported to be higher than 90% [109,110]. There are very few cases of surgical tracing with radioactive tracers alone. Radioisotopes are typically utilized in conjunction with the other dyes, such as ICG or blue dyes, which is considered the gold standard for SLN detection [83,104].

### 3.5. Dual Tracers

In order to solve the problem of lymphatic vessels not being revealed by the radiotracer and the false positives caused by the low molecular weight of dyes, two tracers are often applied in one operation. Injections of radioactive tracers are usually performed one day before surgeries. After a 15 min interval following intraoperative submucosal injections of blue dye or ICG, SLNs are detected with the naked eye and gamma-ray detectors [113,114,115]. Despite the use of ICG, its properties cannot be fully exploited in the absence of near-infrared or fluorescence imaging, which might account for the low efficiency of single-tracer detection in certain studies. The combination of the NIFI properties of ICG with radioisotope detection is only mentioned in one trial protocol [116]. In general, the application of dual tracers improves data describing LN detection since the two complement each another. It was pointed out in one study that the number of hot (high radioactive signal) or dyed nodes was about 2.5 times that of hot and dyed nodes [117]. The detection rate, sensitivity, and accuracy were 91.2%, 100%, and 100% separately, while these data were 73.5%, 72.2, and 94% in the dye group and 80.9%, 83.3%, and 98.2% in the radioisotope group [106]. The accuracy became even higher with the usage of sentinel lymphatic basin detection since some patients showed only lymphatic duct staining, and abdominal fat and the primary tumor also had influences on LN identification [111,112]. With false-negative rates ranging from 0% to over 10%, scientists have attempted to explore the causes of this phenomenon [106,109,118,119,120]. The stage of the tumor appears to be its main predictor [120,121]. Furthermore, the radioisotope count was proved to be associated with LN metastasis since the radioisotope counts for metastatic SLNs, lymphatic stations, and basins were substantially higher than those without metastasis [122].

In addition to conventional combined usage, patent blue and ICG are combined for dual tracing [123]. SLNs were harvested 3 to 5 min after subserosal injection of patent blue and ICG at three points, respectively. It was found that the false-negative rate was much lower than that of single-tracer methods. In our institution, the combination of ICG and CNSI is routinely performed for lymph node dual tracing in gastric cancer surgery. To take full advantage of the properties of CNSI for tumor localization and ICG for LN tracking, our institution has attempted to conduct submucosal CNSI plus subserosal ICG-guided laparoscopic gastrectomy, which ensures more accurate tumor identification and enhanced LN detection number.

### 3.6. Novel Tracers

Regrettably, the primary mechanism of LN localization in traditional tracers depends on their physical properties. Tracers with specific tumor-targeting effects are likely to play a better role. There is one potential tumor-specific tracer, 5-aminolevulinic acid (5-ALA), that is converted to protoporphyrin IX and accumulates in tumors because of changes in enzyme activity in cancer cells [124]. Cumulated protoporphyrin IX thus emits red fluorescence under blue light excitation [125]. Unfortunately, 5-ALA can only be applied for postoperative detection because of the poor penetration of blue light [126]. A carcinoembryonic antigen-targeted fluorescent probe has also been developed for clinical trials [127].

With the advance of nanomedicine, multiple nanoparticles have been utilized in gastric carcinoma diagnosis and treatment preclinically, which is another potential development direction [128]. Through exploitation of the particular combination properties of the Arg-Gly-Asp (RGD) sequence and of integrin overexpressed in gastric cancer cells, ICG has been transported to tumor locations for more specific imaging [129,130]. Cyclic RGD has been demonstrated to direct the precise uptake of nanomedicines by metastatic gastric cancer cells in SLNs as well [131]. Tumor-specific markers, such as HER-2, can also be selected as targets [132]. However, there is still a long way to go for the widespread clinical application of nanomedicines.

## 4. Conclusions and Future Perspectives

The clinical application of tracers has the potential to alter the way that GC surgery is conducted, whether in resection line identification or LN detection. With the widespread use of intraoperative tracers, the applications and indications of minimally invasive surgical approaches may be greatly broadened and improved [133]. Allergic reactions appear to be the most common adverse reactions to tracers. Even so, the reported incidence of blue dye anaphylaxis is only 0.07% to 2.7% [34]. Few fatalities have been reported in allergy sufferers and certain precautions can be taken to prevent allergic responses as well. The allergy rate for new tracers, such as ICG, is lower. In general, the safety of intraoperative tracers is assumed to be guaranteed. Despite the existence of different tracing and imaging principles, diverse traces have shown excellent effects on surgical navigation and huge potential. The advantages and disadvantages of each tracer are shown in Table 1.

Nevertheless, the exploration of clinical tracers still has a long way to go. There are no guidelines clearly specifying the exact use methods, indications, and contraindications of any tracer. Only the Chinese clinical guidelines mention the possible application prospects of tracers [134]. Based on data from several clinical studies, we can only roughly conclude that excessive fat, preoperative chemotherapy, and advanced tumor staging seem to be unfavorable to the functions of tracers [3,25,51,111]. Numerous limitations remain to be addressed. Firstly, there are still common problems, such as the high false-negative rate and shorter-than-expected surgical margins, during tracer application. Moreover, it is not feasible to detect LNs in individuals with advanced tumor stages and obesity. As surgical tracer usage is still in its infancy, more attempts are required to improve tracer experimental skills and clarify indications. Secondly, most of the articles covered in this review were conducted by different surgical teams. The data obtained probably vary due to the diverse methods of utilizing tracers and the discrepant details of the gastrectomies and lymphadenectomies. Therefore, large-scale, prospective, multicenter clinical studies are required to quantify the exact effects of each tracer. Furthermore, the dual-tracer approach seems to be the preferred choice nowadays due to its higher efficiency compared to single-tracer techniques. Rarely have attempts been put into practice, apart from the simultaneous use of radioisotopes and blue dyes or ICG. Additional drug delivery combinations or novel dosing methods could be explored.

In conclusion, tracers have been proven to be safe and effective. Through the refinement of traditional tracer use and the application of novel tracers, gastric cancer surgery may become more refined, reducing patient pain and improving prognosis.

## Figures and Tables

**Table 1:** The pros and cons of different tracers.

Tracers	Advantages	Disadvantages
Blue dye	Direct observation of lymphatic drainage regions and lymphatic vessels; low price; easy availability; tractability	Digitizing challenges; rapid diffusion and degradation; allergic reactions; challenges in detecting LNs in obese patients
ICG	Lower allergic reaction rate; capability to detect LNs beneath adipose tissue; obvious visualization; signal stability	Professional testing equipment required
Carbon particles	Long accumulation time in LNs; convenience; cheapness; wide availability	Possibility of staining the entire surgical area black
Radioactive tracer	Rare allergic reactions; easy to digitize; objectivity; long residence time in SLNs	Unable to monitor lymphatic vessels; expensiveness; radioactivity; lack of visual inspection; high background scattering; legal restrictions; radioisotope scarcity; requirement for advanced instruments and radiation protection
